# Cloning andexpression of cDNA encoding growth hormone tambaqui (*Colossoma macropomum*) in the yeast *Pichia pastoris*

**DOI:** 10.1186/1753-6561-8-S4-P146

**Published:** 2014-10-01

**Authors:** Elson A Sadalla-Pinto, Jorge IR Porto, Suelen D Silva, Edson J Carmo, Edmar V Andrade, Spartaco Astolfi-Filho

**Affiliations:** 1 Universidade Federal do Amazonas/Centro de Apoio Multidisciplinar, Lab. Tecnologias do DNA Recombinante, Manaus-AM, Brazil; 2 Instituto Nacional de Pesquisas da Amazônia/CBIO, Lab. Genética Animal, Manaus-AM, Brazil

## 

Genetic studies involving the search, cloning and expression of genes encoding proteins involved in important physiological processes and advantageous features of the economic standpoint have become the biotechnology research increasingly promising. Due to its zootechnical importance, the encoding gene of growth hormone (GH) of various fish species have been isolated, cloned and expressed in heterologous expression systems. The tambaqui (*Colossoma macropomum*), Amazonian fish species considered promising for fish farming, has been the subject of extensive genetic research with biotechnology focus. This study aimed to express the cDNA encoding GH tambaqui (tGH) in the methylotrophic yeast *Pichia pastoris*. The nucleotide sequence of cDNA tGH, previously isolated by Sousa (2009) [1], was optimized for expression in *P. pastoris *and obtained by chemical synthesis. It was then cloned into the cloning vector pUC19 followed by subcloning into expression and secretion vector pPIC9 with the endonucleases *Eco *RI and *Not*I. The recombinant plasmid named pPIC-tGH was linearized and inserted into host by electroporation. Recombinant clones were selected in medium auxotrophic for histidine. The expression of recombinant protein was induced by methanol addition during 96 hours occurred in shake flasks. The culture supernatant was analyzed on SDS-PAGE gel and expression of tGH was confirmed by Western blotting. The analysis of the supernatant revealed in both phenotype Mut^+ ^and Mut^s ^the presence of a protein band of approximately 23 kDa, which was confirmed to be the tGH recombinant by Western blotting using monoclonal antibody against histidine tail C- terminal. The expression of tGH started already first 24 hours and was sustained throughout the induction period, lasted up to 120 h. These results are similar to of Li et al. [2], who expressed GH carp (*Cyprinus carpio*) in *P. pastoris *and observed that from the first 24 hours of induction the recombinant protein was already present in the medium, and time 72 and 96 hours corresponded to the peak of expression. These results demonstrate that GH tambaqui can be successfully expressed in *P. pastoris*. The tambaqui, for their economic importance, so far was the first Amazonian fish species to have GH expressed in heterologous system, the first step in the production of GHs of other important species for aquaculture regional and national. In the future, tambaqui recombinant GH should be analyzed for their efficiency in accelerating the growth of tambaqui towards the possible use in aquaculture production, which represent progress and innovation in technologies of cultivation of fish Amazonian.
